# Risk factors of recurrence for resected T1aN0M0 invasive lung adenocarcinoma: a clinicopathologic study of 177 patients

**DOI:** 10.1186/1477-7819-12-285

**Published:** 2014-09-13

**Authors:** Fan Yang, Kezhong Chen, Yida Liao, Xiao Li, Kunkun Sun, Dongmei Bao, Jun Wang

**Affiliations:** Department of Thoracic Surgery, Peking University People’s Hospital, 11 Xizhimen Nan Ave, Beijing, 100044 China; Department of Pathology, Peking University People’s Hospital, 11 Xizhimen Nan Ave, Beijing, 100044 China

**Keywords:** Lung adenocarcinoma, Recurrence, New classification, Solid predominant adenocarcinoma, Micropapillary predominant adenocarcinoma

## Abstract

**Background:**

This study aimed at identifying risk factors of recurrence for completely resected pathologic T1aN0M0 lung adenocarcinomas.

**Methods:**

We reviewed the records of 177 T1aN0M0 invasive adenocarcinoma patients, and re-classified achieved surgical specimens according to the new International Association for the Study of Lung Cancer, American Thoracic Society, and European Respiratory Society (IASLC/ATS/ERS) lung adenocarcinoma classification. Impact on recurrence-free survival (RFS) for age, gender, smoking history, lymphovascular invasion (LVI) and new classification was analyzed by log-rank test and Cox regression. Two existing prognostic grouping schemes of new classification were compared, and subsequently, the correlation of high-grade group in the better prognostic grouping model with clinical data was investigated statistically.

**Results:**

The 5-year recurrence-free rate was 83.7%. The LVI and new adenocarcinoma classification were significantly associated with 5-year RFS (*P* = 0.012; *P* = 0.022, respectively). The designation of papillary predominant subtype in the low-grade group, along with lepidic- and acinar predominant subtype had more prognostic significance than the model of combining papillary-, solid- and micropapillary predominant subtypes as the high-grade group (*P* = 0.005 versus *P* = 0.181). This high-grade group has increased risk of recurrence in a multivariate Cox regression (adjusted HR 2.815, 95% CI: 1.239 to 6.397, *P* = 0.013), and is associated significantly more with male gender (adjusted OR 2.214, 95% CI: 1.050 to 4.668, *P* = 0.037), and, with borderline significance, the presence of LVI (adjusted OR 2.091, 95% CI: 0.938 to 4.662, *P* = 0.071).

**Conclusions:**

Our results showed that the solid- and micropapillary predominant subtype of IASLC/ATS/ERS classification remains the only risk factor for post-operative recurrence of T1aN0M0 adenocarcinomas, suggesting that they can be indicators of aggressive tumor behaviors.

## Background

Lung carcinoma is one of leading causes of cancer death worldwide, and its incidence rate continues to increase [1]. Adenocarcinoma is the most common histologic type of lung cancer, especially among Chinese women [2]. The widespread use of computed tomography (CT) screening encouraged by positive results of the National Lung Screening Trial is detecting more and more early-staged adenocarcinomas [3, 4]. T1aN0M0 constitutes the earliest stage of invasive lung cancer. As the standard of care, follow-up and surveillance without adjuvant therapy are recommended after complete resection [5]. However, some patients experience recurrence and die of lung cancer. According to the seventh lung cancer staging project by the International Association for the Study of Lung Cancer (IASLC), T1a non-small cell lung cancer (NSCLC) has a 5-year survival of about 80% [6]. Therefore, there has been a need to identify these small but aggressive tumors for more careful follow-up and/or adjuvant therapy.

Many efforts have investigated the use of clinical, radiologic, and histologic factors to refine the prognosis of adenocarcinomas after surgical resection. Studies have reported prognostic significance of gender [7, 8], smoking history [9], tumor size [10], degree of differentiation [11], visceral pleural invasion [12], lymphovascular invasion (LVI) [13, 14], ground-glass opacity/solid ratio [15], size of invasive tumor [16], maximum uptake on positron emission tomography (PET) scan [10, 15]. Given the fact that prognostic factors of lung cancer may vary with tumor size [17], whether these factors have prognostic value in T1aN0M0 adenocarcinomas is still unknown.

Remarkable advances in understanding lung adenocarcinoma have led to a new classification system, sponsored by the IASLC, the American Thoracic Society (ATS), and the European Respiratory Society (ERS) [18]. Several independent studies have validated the prognostic significance of new adenocarcinoma histologic subtypes [16, 19, 20], but little attention has been paid to T1a tumors after radical resection. However, previous studies of adenocarcinoma ≤ 2 cm failed to include new classification into the analysis [13, 21, 22]. The prognostic factors for completely resected T1aN0M0 adenocarcinomas are still an open question. It has been suggested that prognostic factors for lung cancer may vary with tumor size [17].

The main purpose of our study was to determine the risk factors for recurrence in a cohort of Chinese patients with radically resected invasive T1aN0M0 adenocarcinoma.

## Methods

### Patients

This study was approved by the Institutional Review Board of Peking University People’s Hospital. Consent from patients was waived as this study is retrospective and no personal identifiable information was included in the manuscript. We performed a retrospective review of a prospectively maintained lung cancer database of all surgically resected NSCLC patients at our institution from January 2004 to September 2011. The inclusion criteria included complete resection by lobectomy with mediastinal lymph node dissection and a definitive pathologic confirmation of T1aN0M0 lung adenocarcinoma. Those patients with preinvasive or minimally invasive lesions defined by the new classification [18], or concurrent malignancy, or multiple tumors were excluded. Follow-up was performed on an outpatient basis at three-month intervals for the first two years and at six-month intervals thereafter. In all, 177 patients were included in this study.

### Parameters and histologic subtyping

Data extracted from each patient’s medical record included age, sex, smoking history, tumor size and tumor grading. Maximum uptake on PET scan was not chosen, because it is size-dependent and there is controversy regarding the value of PET scanning for peripheral clinical T1a lesions [23]. Measurements for percentage of ground-glass opacity (GGO), which are proposed as an indicator for less invasive tumor [15], were not included in the present analysis.

All formalin-fixed surgical specimens were categorized according to the IASLC/ATS/ERS classification of lung adenocarcinoma [18]. Two pathologists reviewed all H&E-stained slides, recording the percentage of each histologic component in 5% increments, and discussing until consensus was achieved. The predominant subtype was defined as the pattern found with the largest proportion [18]. The presence of lymphovascular invasion (LVI) was defined as the presence of aggregates of tumor cells inside vascular or lymphatic micro-vessels.

### Endpoint

In the current study, recurrence-free survival (RFS) was defined as time interval from surgery to the time of diagnosis of first definite clinical or pathologic evidence of local recurrence or metastatic disease, lung cancer-related death, or last follow-up. Patients were censored if they were alive with recurrence-free status at the time of the most recent follow-up or had died without documented recurrence. Overall survival was not an endpoint for this study. The interest comes from the success of epidermal growth factor receptor (EGFR) inhibitors, which improve the median survival of late-staged EGFR mutation-positive patients from about ten months with chemotherapy alone [24], to over thirty months [25]. Prognostic analysis of overall survival will be biased by predictive factors, such as EGFR mutation, which associates with female sex and the status of having never smoked [26].

### Statistical analysis

The RFS was plotted by the Kaplan-Meier method, and differences in RFS were assessed using the log-rank test. Risk factors associated with RFS in univariate analysis with Cox proportional hazards model analysis with a *P*-value less than 0.10 were entered into a multivariate analysis. Correlation analysis of clinicopathologic variables with histologic subtype groups was performed by Logistic regression. A *P*-value less than 0.05 was considered statistically significant. Statistical calculations were performed using SPSS (version 16.0; SPSS, Chicago, IL, USA).

## Results

### Patient characteristic

Of the one hundred and ninety-eight patients who fulfilled the inclusion criteria, eight patients were excluded due to preinvasive adenocarcinoma, eight patients due to multiple tumors and five patients due to concurrent malignancy during the past five years. Finally, 177 patients were included in this study. The clinicopathologic features including age, gender, smoking history, tumor size, LVI and new IASLC/ATS/ERS classification are listed in Table 1. More patients were female (98 patients, 55.4%) and the median age was 65 years (range 82 to 33 years). The median follow-up period was 44 months (range 7 to 89 months).

**Table 1:** **Clinicopathologic characteristics of 177 patients with invasive T1aN0M0 adenocarcinoma**

Variables	Number (%)	Five-year RFS%	***P***-value by log-rank test
Age			
≤ 65	97	83.7%	0.520
> 65	80	83.5%	
Sex			
Male	79	84.1%	0.855
Female	98	83.4%	
Smoking history			
Never-smoked	128	81.7%	0.424
Smoker	49	88.7%	
Tumor size			
≤ 1 cm	21	95.2%	0.231
> 1 to ≤ 2 cm	156	82.2%	
LVI			
Absent	135	87.5%	0.012
Present	42	72.1%	
Histological grading			
Well differentiated	78	85.4%	0.535
Moderately differentiated	66	82.0%	
Poorly differentiated	33	83.0%	
Predominant subtypes			
Lepidic	45	94.9%	0.022
Acinar	58	83.1%	
Papillary	37	91.9%	
Solid	18	67.4%	
Micropapillary	19	57.6%	

*Abbreviations*: *LVI* lymphovascular invasion, *RFS* recurrence-free survival.

All specimens were confirmed as invasive adenocarcinomas and then re-classified according to the criteria of the IASLC/ATS/ERS classification of lung adenocarcinoma [18]. The most frequent histologic subtype in our cohort was acinar predominant, consisting of 58 cases (32.8%) followed by lepidic predominant, comprising 45 tumors (25.4%). Papillary-, micropapillary-, and solid predominant adenocarcinomas accounted for 37 (20.9%), 19 (10.7%), and 18 (10.2%) specimens respectively (Table 1). Variant adenocarcinomas were not found.

### Survival analysis

**Figure 1:**
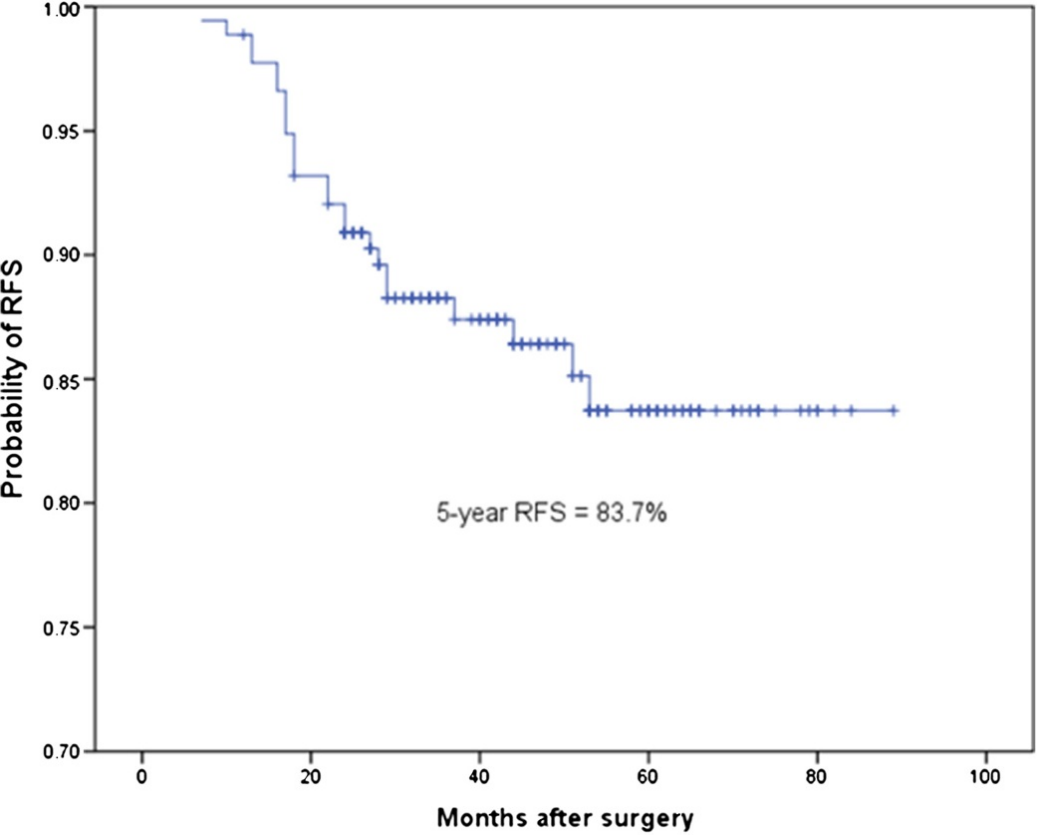
**Recurrence-free survival (RFS) of all 177 patients with invasive T1aN0M0 lung adenocarcinoma.**

During the median follow-up period, 24 recurrences were documented, including 2 local recurrences and 22 distant metastases (with/without local recurrence). Of these recurrences, ten were confirmed pathologically, mostly by fine-needle aspiration or supraclavicular lymph node biopsy, and the remainder was defined by radiologic evidence. Five-year RFS was 83.7% (Figure 1). Log-rank tests of each parameter showed that the presence of LVI was a significant predictor of 5-year RFS (72.1% versus 87.5%, *P* = 0.012). Adenocarcinoma classification was also significantly associated with risk of recurrence (*P* = 0.022). Lepidic predominant subtype had the highest 5-year RFS (94.9%), followed by papillary predominant (91.9%). The micropapillary predominant subtype had the lowest RFS (57.6%), followed by the solid predominant (67.4%). The acinar predominant subtype had a 5-year RFS of 83.1% (Table 1).

**Figure 2:**
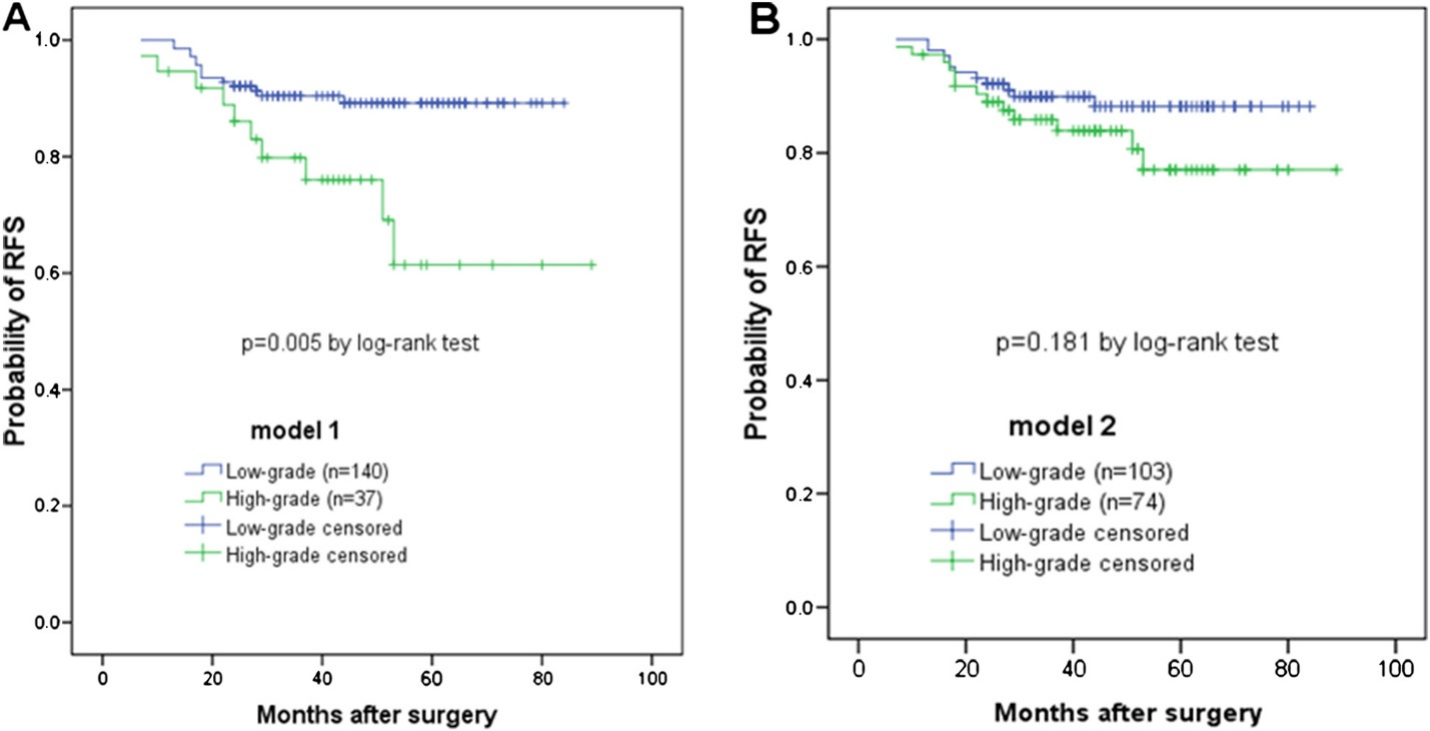
**Probability of recurrence-free survival of different histologic prognostic groups. (A)** In grouping scheme 1, the papillary predominant subtype was designated in the low-grade group, along with lepidic- and acinar predominant subtypes. The low-grade group had significantly lower probability of recurrence (*P* = 0.005). **(B)** Grouping scheme 2 combined papillary-, solid- and micropapillary predominant subtypes as the high-grade group. The difference of probability of RFS between the low- and the high-grade groups failed to reach statistical significance (*P* = 0.181).

In order to increase statistical power, we combined predominant subtypes with similar survival into groups, as was done in previous studies [16, 19, 27–29]. However, the grouping schemes differ on the designation of papillary predominant tumors; whether it should be grouped as high-grade [7, 27, 29] or intermediate-grade [16, 19, 28, 30, 31]. We compared both grouping schemes on our cohort. For model 1, the low-grade group included lepidic-, acinar-, and papillary predominant adenocarcinomas, while the high-grade group consisted of solid- and micropapillary predominant tumors. Regarding model 2, the papillary predominant was designated also as the high-grade group. Log-rank tests of both models showed designation of the papillary predominant into the low-grade group better stratified prognosis with respect to 5-year RFS (*P* = 0.005 for model 1 versus *P* = 0.181 for model 2) (Figure 2). Model 1 of grouping was used for further analysis in this study.

We then performed Cox multivariate survival analyses. We included those factors that were significant in univariate survival analysis, namely, sex, LVI, and pattern groups. The analysis showed that the adenocarcinoma subtype grouping (model 1) remained significantly associated with RFS, with patients in the high-grade group having an increased risk of recurrence, compared with the low-grade group (adjusted hazard ratio (HR) 2.815, 95% CI: 1.239 to 6.397, *P* = 0.013) (Table 2). LVI only showed borderline significance (adjusted HR 2.100, 95% CI: 0.930 to 4.741, *P* = 0.074).

**Table 2:** **Multivariate analysis for five-year recurrence-free survival (RFS)**

Variables	Univariate Cox,***P***	Multivariate Cox, HR (95% CI)	***P***
Age	0.233		
Sex	0.517		
Smoking history	0.428		
Tumor size	0.079	2.974 (0.798 to 11.078)	0.104
LVI	0.016	2.100 (0.930 to 4.741)	0.074
Histological grading	0.362		
Subtype grouping	0.008	2.815 (1.239 to 6.397)	0.013

*Abbreviations*: *CI* confidence interval, *HR* hazard ratio, *LVI* lymphovascular invasion.

### Correlation of more aggressive subtypes with clinicopathologic variables

Finally, we performed correlation analysis to explore the relationship between the clinicopathologic parameters and the adenocarcinoma prognostic grouping by logistic regression. Multivariate regression of significant variables in univariate analysis showed significant association of male sex with high-risk group (adjusted odds ratio (OR) 2.214, 95% CI: 1.050 to 4.668, *P* = 0.037). The statistical significance for LVI was borderline (adjusted OR 2.091, 95% CI: 0.938 to 4.662, *P* = 0.071).

## Discussion

To the best of our knowledge, the current study is the first clinicopathologic prognostic analysis of T1aN0M0, incorporating clinicopathologic and new IASLC/ATS/ERS classification variables [13, 22]. The only published study investigating the prognostic utility of the classification for patients with ≤ 2 cm adenocarcinomas focused on the association of the percentage of micropapillary component with recurrence, and between lobectomy versus limited resection. We carefully designed this retrospective study, trying to identify risk factors that can reflect accurately the behaviors of early-staged lung adenocarcinomas.

Because adenocarcinoma *in situ* (AIS), and minimally invasive adenocarcinoma (MIA) have 100% or near 100% disease specific survival after complete resection [18], they were excluded in our study of risk factors for recurrence. Visceral pleural invasion (VPI) was also excluded since it is not only determined by aggressiveness of tumor, but also by its anatomic location. Although one study which focused on adenocarcinomas < 2 cm found no impact of VPI on recurrence or survival [32], a nation-wide registry study confirmed the association of worse survival with VPI in NSCLC of all sizes [33], and the adverse impact of VPI was hypothesized as dissemination through parietal sub-pleural lymphatic drainage [34]. Moreover, we chose patients after radical lobectomy and lymph node dissection, so that surgical margin-related recurrence would not influence the analysis [35].

Our cohort of T1aN0M0 lung adenocarcinoma patients had a 5-year RFS of 83.7%, similar to previous reports [22, 36]. The distribution of IASLC/ATS/ERS subtypes varies considerably in the literature, which may have resulted from differences between geographical regions and ethnic populations (east versus west) [16, 31], patient groups (all stages versus stage I) [29], and the expertise of the pathologists [37]. The frequency of lepidic predominant ranged from 26.7% reported by a Japanese study to 5.6% in a US cohort, while that for papillary predominant ranged from 40.9% to 4.7%. The percentage of micropapillary ranged from 15.2% to 0 out of 191 adenocarcinomas in a Japanese study. In the current study, the most frequent histologic subtype was acinar predominant, comprising 58 out of 177 cases (32.8%), followed by lepidic predominant, 25.4%. Papillary-, micropapillary-, and solid predominant adenocarcinomas accounted for 37 (20.9%), 19 (10.7%), and 18 (10.2%) specimens respectively.

In common with previous reports [7, 13, 16, 19, 28–31, 38, 39], our data confirmed the prognostic value of the IASLC/ATS/ERS histologic classification, along with LVI, by log-rank test. The 5-year RFS of lepidic predominant was 94.9%; papillary predominant, 91.9%; acinar predominant, 83.1%; solid predominant, 67.4%, and micropapillary predominant, 57.6%. To gain more statistical power, previous studies combined subtype of similar prognosis to construct a two-tiered or three-tiered grading scheme. However, difference remains regarding the designation of papillary predominant adenocarcinomas. Interestingly, most studies on Asian patients grouped this subtype into lower- or intermediate-grade [16, 19, 28, 30, 31], while most studies from western countries, grouped it into the high-grade group [27, 29] except that by Yoshizawa *et al*. [28]. Comparison of these two grouping schemes on our cohort favored the former way of grouping (Figure 2, *P* = 0.005 versus *P* = 0.181), suggesting that the behaviors of papillary predominant subtype may have ethnical differences. This hypothesis can be supported by the findings in Asian patients of the association between EGFR mutation and papillary predominant adenocarcinomas [39, 40], but not in an Australian cohort [27].

The observation that new histologic subtype grouping remained the only risk factor for recurrence in patients of T1aN0M0 disease on multivariate Cox regression, and reported correlation of clinicopathologic variables with histologic subtypes, promoted us to investigate the relationship of these variables with subtype groupings. We chose low-grade or high-grade as binary dependent variables instead of each histologic subtype, for the reason that the present study focused on recurrence risks, which are more significantly related to subtype groupings. The correlation analysis confirmed the association of male gender and presence of LVI (borderline significant) with high-grade group (Table 3). Other researchers have reported similar associations. Hung *et al*. found that the lepidic predominant subtype is associated with less smoking exposure, smaller tumor size, absence of LVI, and well/moderately differentiated histologic grade, while the high-grade group member, solid predominant, is associated with male gender, smoking exposure, larger tumor size, and poorly differentiated histologic grade [19]. Yanagawa and colleagues reported higher frequency of smokers in solid predominant than in other subtypes [16]. Our results, along with these observations, suggested that these clinicopathologic variables, such as gender, smoking history and LVI, might not be independent prognostic factors, which may challenge the independency of these prognostic factors, such as female gender [7].

**Table 3:** **Correlation between clinicopathologic factors and high-grade adenocarcinoma group**

Variables	Univariate logistic,***P***	Multivariate logistic, OR (95% CI)	***P***
Age	0.195		
Sex	0.036	2.214 (1.050 to 4.668)	0.037
Smoking history	0.469		
Tumor size	0.884		
LVI	0.070	2.091 (0.938 to 4.662)	0.071
Histological grading	0.225		

*Abbreviations*: *CI* confidence interval, *LVI* lymphovascular invasion, *OR* odds ratio.

The local infiltration ability and metastatic potential of solid- and micropapillary predominant subtypes in the high-grade group, may explain the increased risk of recurrence. A recently published comparison of small (≤2 cm) adenocarcinoma patients who underwent sublobar resection versus lobectomy found that tumors with ≥ 5% micropapillary component had more locoregional recurrences after limited resection, especially those with small surgical margins, but not in cases who underwent lobectomies [35]. The authors suggested a greater capacity for local infiltration of micropapillary subtype. Likewise, a study of adenocarcinomas with N2 metastasis suggested greater metastatic potential of micropapillary and solid predominant subtypes, even though they may not be the predominant subtype [27]. In a study of adenocarcinoma of post-operative adenocarcinomas at all stages, Russell reported the highest incidence of N2 metastases and invasion of lymphovascular spaces and visceral pleura for micropapillary predominant adenocarcinoma [20]. Indirect evidence also comes from a subtyping analysis of stages I to IV adenocarcinomas, which showed the highest rate of nodal metastases was for micropapillary predominant subtype (76%), followed by solid predominant (51%), whereas for lepidic, the rate was only 7% [29]. However, with respect to prognosis, the predominant subtype was the main determinant [27, 29].

There is growing interest in considering sublobar resection for early-stage lung cancer. To date, limited evidence only supports the use of sublobar resection for subsolid lesions, a radiologic feature of preinvaisve or less invasive tumors [5, 41]. Histologic subtypes of adenocarcinoma according to IASLC/ATS/ERS classification also stratifies invasiveness and may have potential implication for selecting limited resection histologically, as suggested by Nitadori [35]. However, the accuracy of reporting adenocarcinoma subtypes on frozen section is still an open question.

We acknowledge several limitations of our retrospective study, which has a relatively small cohort of patients with sub-optimal follow-up periods. The fact that even indolent ground-glass opacity lesions can develop local recurrence more than five years after resection [42] implied the necessity of very long follow-up for these very early-staged cases. Moreover, not all recurrences were biopsy-confirmed, which is more accurate than radiologic evidence.

## Conclusions

Our results showed that the solid- and micropapillary predominant subtype of IASLC/ATS/ERS classification remains the only risk factor for post-operative recurrence of T1aN0M0 adenocarcinomas, suggesting that they can be indicators of aggressive tumor behaviors. We agree with the proposal by Warth *et al*. that a tumor grading system based on the predominant pattern might assist clinical decision-making for pulmonary adenocarcinoma management [29].

## Authors’ information

FY, KC and XL are staff members of Department of Thoracic Surgery, Peking University People’s Hospital. YL is a PhD student of Peking University Health Science Center. KS and DB are staff members of Department of Pathology, Peking University People’s Hospital.

## Abbreviations

AIS: adenocarcinoma *in situ*
CT: computed tomography
EGFR: epidermal growth factor receptor
GGO: ground-glass opacity
H&E: hematoxylin and eosin
HR: hazard ratio
IASLC/ATS/ERS: International Association for the Study of Lung Cancer, American Thoracic Society, and European Respiratory Society
LVI: lymphovascular invasion
MIA: minimally invasive adenocarcinoma
NSCLC: non-small cell lung cancer
OR: odds ratio
PET: positron emission tomography
RFS: recurrence-free survival
PET: positron emission tomography
VPI: visceral pleural invasion.
